# Increased Reproductive Output and Telomere Shortening Following Calcium Supplementation in a Wild Songbird

**DOI:** 10.1002/ece3.70483

**Published:** 2024-10-27

**Authors:** Marina D. Rodriguez, Susan M. Bailey, Paul F. Doherty, Kathryn P. Huyvaert

**Affiliations:** ^1^ Department of Biology Colorado State University Fort Collins Colorado USA; ^2^ Department of Environmental and Radiological Health Sciences Colorado State University Fort Collins Colorado USA; ^3^ Department of Fish, Wildlife and Conservation Biology Colorado State University Fort Collins Colorado USA; ^4^ Department of Veterinary Microbiology and Pathology Washington State University Pullman Washington USA

**Keywords:** calcium supplementation, cost of reproduction, life‐history tradeoff, telomeres, tree swallows

## Abstract

Life history theory predicts increased parental investment comes with fitness costs, often expressed as negative effects on survival and future reproduction. To better understand the costs of reproduction and life history trade‐offs, we evaluated calcium supplementation at a high‐elevation site in Colorado as a novel approach to experimentally alter reproductive investment in nesting female *Tachycineta bicolor* (tree swallow). Calcium is a nutrient critical to avian reproduction as the intake of natural calcium is essential for egg production, embryo development, and nestling growth. Altering calcium availability exclusively during the breeding season allowed examination of individual biological responses to experimental modification of reproduction, as well as the reproductive costs associated with egg production and laying an entire clutch. As a functional endpoint and proxy for fitness and longevity, telomere length was measured at the beginning and end of each breeding season. Telomeres—protective “caps” at the ends of chromosomes—have been shown to shorten with aging and a variety of stressors, including higher reproductive output. Results demonstrate that tree swallow mothers supplemented with calcium during the breeding season experience higher reproductive output and produce offspring with longer telomeres, which came at the cost of relatively shorter telomeres during the reproductive season. These findings provide additional support for reproductive trade‐offs, and also challenge previous calcium supplementation studies that suggest excess calcium reduces the cost of reproduction.

## Graphical abstract

We used calcium supplementation in nesting *Tachycineta bicolor* (tree swallow) and measured telomere length before and after breeding to better understand the costs of reproduction and life history trade‐offs. We found that mothers supplemented with calcium had higher reproductive success and greater telomere shortening, though their offspring had longer telomeres at 12 days old.

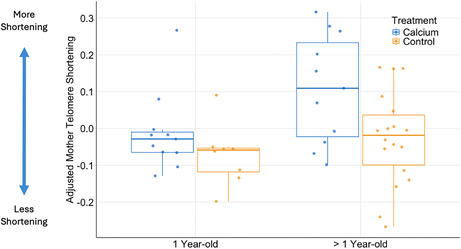

## 1 Introduction

Life history theory is built upon the premise that resources, and the time and energy it takes to acquire them, are limiting, and that allocation of these resources to competing functions can result in trade‐offs (Stearns 1992). Critical to the evolution of life history strategies is the cost of reproduction, where energy and resources allocated to breeding activities are not available for self‐maintenance. Accordingly, an increase in reproductive investment can be linked to a reduction in longevity through a decrease in investment toward self‐maintenance (Williams 1966; Stearns 1992; Roff 2002). Here, using telomere shortening as a proxy for fitness and longevity, we investigated the cost of reproduction in a wild song bird.

Telomeres are highly conserved repetitive DNA sequences that cap the physical ends of eukaryotic chromosomes, protecting them from degradation and loss (Blackburn 1991). Because conventional DNA polymerases cannot completely replicate linear DNA (the end‐replication problem), telomeres shorten with cell division, and thus with aging (Olovnikov 1996). Telomere length varies among individuals of the same age depending on genetic background (Asghar, Hasselquist, and Bensch 2011; Horn et al. 2011; Pepke et al. 2022), as well as with environmental and social stressors that contribute to cellular oxidation (Chatelain, Drobniak, and Szulkin 2020; Gil et al. 2020; Burraco, Lucas, and Salmón 2022). Variation in telomere length has been correlated with survival in numerous wild animal populations, as individuals with relatively short telomeres or a higher rate of telomere shortening tend to have a higher risk of mortality and concomitant reductions in lifespan across many species (Boonekamp et al. 2013; Vedder et al. 2022; Wilbourn et al. 2018; Whittemore et al. 2019). Due to their connection to survival and lifespan, relative telomere length (RTL) and telomere length dynamics (changes over time/longitudinal analyses) have been used as proxies for fitness in a variety of taxa as well, including birds, mammals, and reptiles (e.g., Fairlie et al. 2016; Fitzpatrick et al. 2021; Le Vaillant et al. 2015; Pauliny et al. 2006; Salmón et al. 2017; Angelier et al. 2013; Boonekamp et al. 2013; Wood and Young 2019). However, conclusions from cross‐sectional studies of telomere length can be confounded by measuring telomere length in older individuals, as disappearance of individuals from a population may not be independent of telomere length (Sudyka et al. 2016). Selective disappearance is therefore an important consideration since age‐related telomere shortening shows significant individual variation.

Recent studies demonstrate that the effort invested in reproduction can contribute to telomere shortening (e.g., Bauch, Becker, and Verhulst 2013; Bichet et al. 2020; Sudyka et al. 2019). However, these studies were either cross‐sectional in design or based on annual measurements of telomere length over consecutive breeding seasons, both of which may prove misleading as assessing telomere length over an entire year cannot be linked directly to the reproductive period of interest. Thus, our first objective was to investigate whether biologically relevant differences in reproductive investment influence telomere shortening specifically during the breeding season. Our second objective was to determine if/how telomere length of offspring might be affected by variation in reproductive success of their mothers, as variation in telomere length during early life has been shown to have long‐lasting effects on fitness (e.g., Eastwood et al. 2019; Heidinger et al. 2021, 2012). Lastly, and consistent with the Terminal Investment Hypothesis (Clutton‐Brock 1984), we sought to test whether older individuals invest more into current reproduction compared to younger individuals as reflected by increased telomere shortening, and in contrast to younger birds who invest more in self‐maintenance to enhance their probability of future reproduction and survival (Godfray, Partridge, and Harvey 1991; Klomp 1970).

To investigate whether variation in reproductive investment influences telomere length in tree swallow mothers and offspring, we employed calcium supplementation at a high‐elevation study site to experimentally modify reproductive output. Calcium is essential for egg production, as well as embryo and nestling skeletal development (Balkan, Karakas, and Biricik 2006); however, the diets of insectivorous and granivorous birds tend to contain inadequate amounts of calcium (Graveland and Van Gijzen 1994). Supplementing calcium for breeding birds in calcium‐depleted areas leads to an increase in reproductive success in the form of larger and more eggs produced, as well as higher hatching and fledging success (Dawson and Bidwell 2005; Graveland and Drent 1997; Mänd and Tilgar 2003; Mänd, Tilgar, and Leivits 2000a, 2000b; Tilgar, Mänd, and Leivits 1999; Tilgar, Mänd, and Mägi 2002). In areas where calcium is readily available, avian reproduction may still be limited by calcium due to naturally occurring variation in environmental conditions that make calcium‐specific foraging more costly, such as the harsh weather at the start of the breeding season—a typical occurrence at our high elevation study site in Colorado. We chose calcium supplementation over clutch enlargement methods, as supplementing mothers with calcium facilitates interrogation of differences in individual biological response to the experimental modification of reproduction regardless of the measure (e.g., egg size, number of eggs, etc.). Calcium supplementation also allows accounting for the reproductive costs associated with egg production and laying an entire clutch.

To confirm that calcium supplementation positively influenced reproductive parameters at our study site, we first conducted a 4‐year calcium‐supplementation trial with female tree swallows. In the two subsequent breeding seasons, we used calcium supplementation to modify reproductive output. Blood samples were collected from mothers immediately before egg‐laying and shortly before fledging to determine the direct relationship between reproductive investment and telomere shortening in our study species. The results of our investigation, carried out in a natural ecosystem, represent an important contribution to the knowledge of life‐history and telomere dynamics in vertebrates.

## 2 Materials and Methods

### 2.1 Study System

We studied female *Tachycineta bicolor* (tree swallows), insectivorous passerine birds that nest readily in human‐made nest boxes (Robertson and Rendell 1990). A typical clutch size is four to seven eggs, and incubation lasts 14–15 days (Stocek 1970). Once hatched, both parents provide care for the altricial nestlings until fledging, which takes 17–23 days (Stocek 1970). Numerous telomere studies have used tree swallows as a wild study species (e.g., Belmaker et al. 2018; Haussmann et al. 2003; Haussmann, Winkler, and Vleck 2005; Ouyang et al. 2016), and the short lifespan of tree swallows enables detection of changes in telomere length more quickly than long‐lived organisms (Haussmann et al. 2003). Studying a species that uses nest boxes also comes with certain logistical advantages, such as the ability to trap breeding females and offspring readily and repeatedly. Ninety (90) 5"×5"×8" pine nest boxes were placed at our high‐elevation study area during each of the first 4 years of the study, the number of which increased to 200 nest boxes for the last 2 years. Although 110 nest boxes were added for the 2017–2018 breeding season, an increase in the number of nests used by tree swallows over those years was not observed.

The calcium supplementation study took place over six summers (2013–2018), while telomere length was measured only during the summers of 2017 and 2018. Our study site was located at the Colorado State University Mountain Campus in Larimer County, Colorado, USA (N40.5611, W105.5978). The selected site was within a mountain valley at an elevation of 2750 m, the highest elevation of any avian calcium supplementation study to date. To date, the area has not been affected by anthropogenic acid deposition and natural sources of calcium are available (Binkley et al. 2003; Clow and Sueker 2000; Mast et al. 2011). The nest box trail runs along the edge of a riparian area and consists of nest boxes mounted on *t*‐posts, ~1.5 m above the ground and at a distance of at least 10 m between boxes.

### 2.2 Study Design and Sampling

Starting in early May during the years of 2013–2018, tree swallow nest boxes were checked daily for the start of nest construction. Once nest initiation was observed, indicated by a shallow grass ring at the base of the nest box, the nest box was randomly assigned to either the calcium supplemented group or the non‐supplemented control group. When initiation of the next nest was spotted, that nest box was assigned to the other group, and so on. Also, during group assignment, females were captured at the nest by covering the entrance once she was inside, or by using a mesh trap door. Once in hand, females were banded and tarsus length (cm), wing length (cm), and mass (g) measured. Birds were then classified into age categories of 1‐year old or greater than 1 year depending on the presence or absence of gray/dull plumage around the beak and eyes (Pyle 1997). After taking physical measurements, blood samples were taken using brachial venipuncture; between 10 and 30 μL blood was collected using an insulin syringe and a 27‐gauge needle as suggested by Owen (2011). Blood was transferred to a heparinized capillary tube and was stored at −20°C until analysis in the laboratory (Criscuolo et al. 2009).

Following capture and depending on the group assignment, nests were either calcium‐supplemented with oyster shell, (commercially prepared for poultry), or with non‐supplemented local soil (control) in a tray attached to the roof of the nest box. The oyster shell was crushed into fragments of a comparable size to that of grit normally consumed by tree swallows (Mayoh and Zach 1986). Oyster shell has been used in previous calcium supplementation studies (e.g., Johnson and Barclay 1996, Bidwell and Dawson 2005), and is similar in composition to snail shells, which are a natural source of calcium (Dawson and Bidwell 2005). Although we had no way of definitively determining whether mothers or offspring actually consumed the supplemented calcium, evidence of supplemented calcium fragments was found in the nests of supplemented birds.

Once laying began, nests were checked daily to determine clutch completion; once complete, individual egg length and width measurements were taken. Egg volume was calculated using the formula *V* = 0.51*LW* ^2^, where *L* is the length of the egg, *W* is the width of the egg, and 0.51 is a species‐specific constant (Hoyt 1979). Once hatched, hatching success was calculated for each nest as the proportion of eggs in the clutch that hatched. Sample sizes differ for each analysis due to missing egg volume measurements before hatching and missing hatching success measurements before fledging. For mean clutch size, 107 calcium supplemented nests were compared to 116 control nests. For mean egg volume per clutch, 88 calcium‐supplemented nests were compared to 93 control nests. For mean hatching success, 105 calcium‐supplemented nests were compared to 112 control nests.

In 2017 and 2018, blood samples were collected from mother birds and offspring when chicks were 12 days old using the same collection and storage methods described above; chicks were also banded at this time. All birds were handled and sampled under a Federal Bird Banding permit from the USGS Bird Banding Laboratory and in accordance with approved guidelines of the Institutional Animal Care and Use Committee of Colorado State University (Protocol # 17‐7304A). For analyzing female tree swallow telomere length over the breeding season, 22 calcium‐supplemented and 26 control birds were sampled before and after breeding. Of the females measured, 29 were 1 year‐old and 19 were older than 1 year. For determining mean offspring telomere length per nest, 18 calcium‐supplemented and 21 control nests were used.

### 2.3 Telomere Length Measurement

DNA was isolated from whole blood samples using the DNeasy Blood and Tissue kit (Qiagen, Valencia, California) following the manufacturer's protocol. DNA purity and concentration was determined using a NanoDrop 8000 spectrophotometer (Thermo Scientific). The average ratio of absorbance at 260 nm over 280 nm was used to check for protein contamination and the average ratio of absorbance at 260 nm over 230 nm was used to check for salt contamination. If either ratio of absorbance was < 1.8, the extract was excluded from further analysis (Morinha, Magalhães, and Blanco 2020). DNA integrity was visually assessed on an agarose gel as recommended by Seeker et al. (2016). Following the protocol of Criscuolo et al. (2009), quantitative Polymerase Chain Reaction (qPCR) was used to quantify telomere length in blood. Telomere length was determined as the ratio (*T*/*S*) of telomere repeat copy number (*T*) to a control single gene copy number (*S*), which was then standardized to a reference sample and expressed as RTL. The primers used to amplify the telomere region were as follows: Tel1b (5′‐CGGTTTGTTTGGGTTTGGGTTTGGGTTTGGGTTTGGGTT‐3′) and Tel2b (5′‐GGCTTGCCTTACCCTTACCCTTACCCTTACCCTTACCCT‐3′). Glyceraldehyde‐3‐phosphate dehydrogenase (GAPDH) was selected as the single‐copy gene; the primers used for amplification of GAPDH were specific GADPH‐F (5′‐GGTAGATGGGAGTTCAGTTGTG‐3′) and GAPDH‐R (5′‐AGAAACAAAGCACTGTCAGGG‐3′). Multiplexed qPCR was used with 3 μL of sample DNA at 3 ng/μl, Tel1b/Tel2b primers at a concentration of 900 nM, and GAPDH‐F/GAPDH‐R primers at a concentration of 400 nM in a final volume of 25 μL containing 10 μL of GoTaq qPCR Master Mix (Promega, Madison, Wisconsin, USA). Cycling conditions for the telomere qPCR were as follows: 10 min at 95°C, followed by 30 cycles of 15 s at 95°C, 30 s at 54.5°C, and 30 s at 72°C. For GAPDH amplification, cycling conditions were as follows: 10 min at 95°C, followed by 40 cycles of 15 s at 95°C, 30 s at 60.5°C, and 30 s at 72°C. DNA samples were run in triplicate and a reference sample was run on every plate to compare measurements between plates. QPCR plates included serial dilutions (0.2, 0.4, 2, 10, 30, and 50 ng) of DNA from the same reference bird to create a reference curve to control for amplifying efficiency of the qPCR. All plates had standard curves with *R* ^2^ > 0.98, as recommended by Morinha, Magalhães, and Blanco (2020). A tree swallow blood sample collected at our study site (but not included in our study) was used as the reference sample. Coefficient of determination was > 0.98 and efficiencies within 100 ± 10% (Telomere standard curve: mean = 1.99, standard deviation = 0.01; GAPDH standard curve: mean = 1.94, standard deviation = 0.01). Within sample triplicates, if coefficient of variation (CV; repeatability) was > 0.14 for a sample *T*/*S* value, one of the triplicates was dropped. Samples were excluded if one of the remaining sample duplicate CVs was still > 0.14. Average repeatability of *T*/*S* values was 0.037 across all samples, and was 0.034 for reference samples. Reproducibility for samples run on multiple plates was measured as the standard deviation of cycle threshold (Cq) values for those samples. Reproducibility for telomeric values was 1.68, while reproducibility for GAPDH was 1.58.

### 2.4 Statistical Analysis

All statistical analyses were performed in R 3.5.2. The “lm” function was used in baseR and the “lmer” function in the “lmer4” package (Bates et al. 2015) to fit linear models and linear mixed models to test for effects of calcium supplementation on reproductive traits, telomere shortening, and telomere length on breeding tree swallows and their offspring. Normality and heteroscedastity assumptions were checked visually by plotting residuals of models. An information‐theoretic approach for model selection and ranking (Burnham and Anderson 2002) was used for each model set using the package MuMin in R (Barton and Barton 2015). A model of no effect was considered in each model set and we considered the model with the lowest AICc value in each set to be best supported by the data (Burnham and Anderson 2002; Burnham, Anderson, and Huyvaert 2011).

We first analyzed egg volume, hatching success, and brood size in relation to calcium treatment to determine whether these reproductive parameters were influenced by calcium supplementation. As clutch and brood size are highly correlated (*r* = 0.66), only clutch size was used in our analysis. Linear mixed effects models for each of the three response variables of interest were constructed, which included “box” as a random effect in each model, and each candidate model set included a treatment effect model (calcium or control), a year effect model, an additive model including treatment and year, an interaction model for treatment and year, and an intercept‐only model indicating no effects. Since the study spanned 6 years, during which time weather conditions at our site varied considerably, a year effect model was also included, as was an additive model for treatment and year to account for possible compounding effects of annual variation in weather conditions and calcium supplementation, and an interaction effect to account for the influence of calcium availability varying by year.

An information‐theoretic approach was used for model selection and ranking for each of the three model sets (Burnham and Anderson 2002). The model with the lowest AICc value in each set was considered best supported by the data. The ∆AICc (difference between each model, w_e_, and the top‐ranking model) and Akaike weights (*w*_*i*_; estimates of the probability that i is the best model given the data and the model set) were also calculated. AICc model selection was also used to analyze samples collected only during the 2017–2018 breeding seasons to ensure differences in reproductive performances between calcium and control nests during the years that telomere measurements were taken.

To measure change in RTL of the mother during the breeding season, we calculated *D*, a measure of temporal RTL shortening adjusted for the regression to the mean, following Kelly and Price (2005) using the equation:

$$D=\rho \left(X_{1}-{\bar{X}}_{1}\right)-\left(X_{2}-{\bar{X}}_{2}\right),$$

where

$$\rho =\frac{2rs_{1}s_{2}}{s_{1}^{2}+s_{2}^{2}},$$

*X*_1_ is the RTL for mothers at time‐point one (pre‐breeding), *X*_2_ is the RTL for mothers at time‐point two (when chicks were 12 days‐old), *r* is the correlation between *X*_1_ and *X*_2_, *s* is standard deviation, and *s* ^2^ is variance. RTL measurements at both time‐points were transformed to a log‐normal scale to achieve normality. We then fit *D* as the response variable in linear mixed effects models and included RTL for mothers at time‐point one as a covariate in every candidate model to account for baseline telomere length. We also included qPCR plate as a covariate in every candidate model to account for differences in repeatability between plates.

To analyze telomere length dynamics in mother tree swallows, a global model consisting of variables that could be influencing telomere length was constructed. These predictor variables included treatment, age, clutch size, and year. As all of our predictor variables have potential for additive effects, all combination of predictor variables included in the global model were compared and ranked based on AICc. Two‐way interactions were only included in the global model if such relationships were considered plausible a priori. The same models were also used when replacing clutch size with brood size to examine whether there was a difference between these two variables.

Average chick RTL per nest at Day 12 was also analyzed using linear mixed effect models. A global model consisting of variables that could be influencing chick RTL was constructed. These predictor variables included treatment, mother RTL at time‐point one, mother age, and year, along with “nest ID” as a random effect included in each model. As all of our predictor variables have potential for additive effects, all combination of predictor variables included in the global model were compared and ranked based on AICc. Again, two‐way interactions were only included in the global model if such relationships were considered plausible a priori.

## 3 Results

### 3.1 Calcium Supplementation and Reproductive Parameters

**FIGURE 1:**
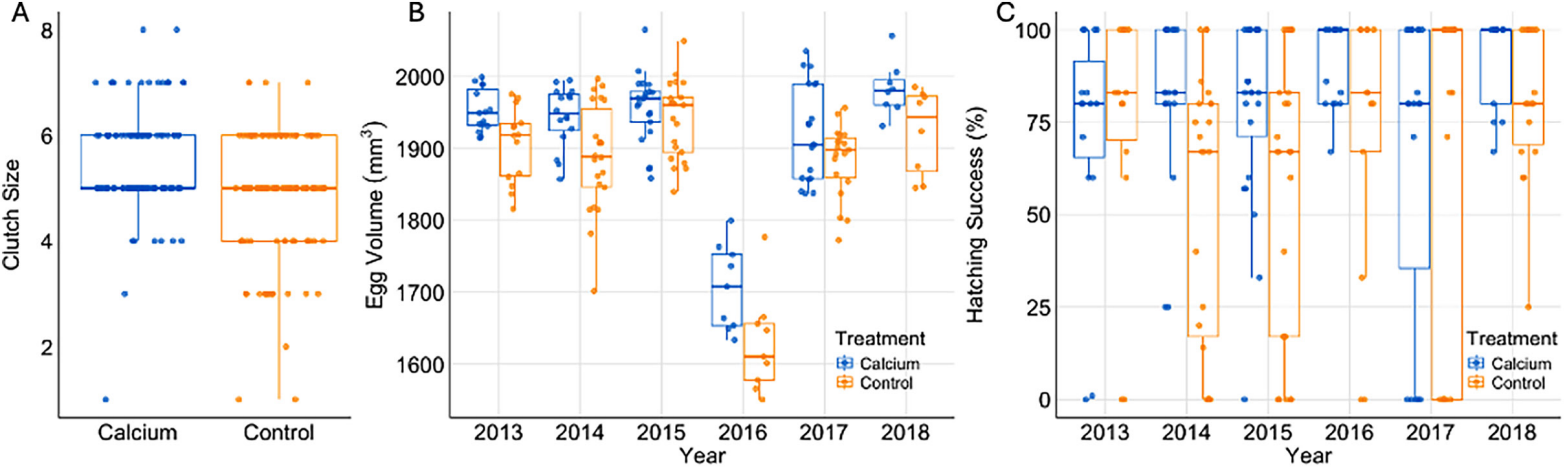
Female tree swallows supplemented with calcium laid and hatched more, larger eggs than those in control groups with some variation over years, as shown by: (A) Tree swallow clutch size per nest of calcium supplemented (*n* = 107) compared to control nests (*n* = 116); (B) tree swallow clutch size per nest of calcium supplemented (*n* = 88) compared to control nests (*n* = 93); and (C) tree swallow hatching success of calcium supplemented (*n* = 105) compared to control nests (*n* = 112) over the course of the 6 years of the study at a high elevation site in northern Colorado.

Two‐hundred and twenty‐four nests were initiated and monitored over the six‐year timeframe of the study. Sample size varied among years, as well as for each analysis, due to differences in nest survival over the various breeding seasons. Only a few individuals were captured multiple times over the 6‐year period, and only the first capture of these individuals was used in the study in order to maintain independence. In analyzing clutch size, the “treatment” model was ranked highest and carried the highest model weight (*w*_*i*_ = 0.80; Table 1A): calcium supplemented nests had statistically significantly larger mean clutch sizes compared to control nests (*p* = 0.002; Figure 1A and Table 2A). Specifically, clutch size in the control nests was estimated to be smaller by 0.59 eggs (95% CI: −0.97, −0.22) compared to the calcium supplemented nests. In analyzing egg volume, the additive “treatment + year” model ranked highest and carried all the model weight (*w*_*i*_ = 1.0; Table 1B); supplemented nests had significantly higher mean egg volumes compared to control nests (*p* < 0.001; Figure 1B and Table 2B). In particular, egg volume in control nests was estimated to be 44.51mm^3^ (95% CI: −22.35, −66.66) smaller than those in calcium supplemented nests. Finally, in analyzing hatching success, the additive “treatment + year” model carried the highest model weight (*w*_i_ = 1.0; Table 1C); a significantly higher proportion of eggs hatched in supplemented nests compared to control nests (*p* = 0.002; Figure 1C and Table 2C). Specifically, hatching success in control nests was estimated to be 13.91% (95% CI: −22.45, −5.36) lower than that of calcium supplemented nests.

**TABLE 1:** Mothers at nests with supplemented calcium produced and hatched more and larger eggs, with some variation by year, as indicated by the model set and rankings exploring the importance of calcium supplementation (“Treatment”) and “year” on mean clutch size per nest, mean hatching success per nest, and mean egg volume per nest in Tree Swallows at a high‐elevation valley in northern Colorado during 2013–2018. The number of parameters (*K*), −2 log‐likelihood (−2LL), and model weights (*w*_*i*_) are shown for each model and the models are ranked by their AICc differences relative to the best model in the set (*∆*AICc_*i*_). The minimum AICc value for mean clutch size per nest was 649.72, for mean egg volume per nest it was 2260.40, and for mean hatching success per nest it was 2296.42. “Box” was included as a random effect in each model.

Dependent variable	Model	*K*	*∆*AICc_*i*_	−2LL	*w*_*i*_
(A) Mean clutch size per nest	Treatment	4	0.00	−315.14	0.80
	Treatment + year	9	3.59	−311.60	0.13
	Intercept	3	5.40	−318.88	0.05
	Treatment × year	14	10.28	−309.36	0.00
	Year	8	11.26	−316.52	0.00
(B) Mean egg volume per nest	Treatment + year	9	0.00	−1075.09	1.00
	Treatment × year	13	31.76	−1095.54	0.00
	Year	8	37.61	−1099.58	0.00
	Treatment	4	75.74	−1122.95	0.00
	Intercept	3	82.01	−1127.13	0.00
(C) Mean hatching success per nest	Treatment + Year	9	0.00	−1093.98	1.00
	Treatment × Year	13	31.71	−1114.39	0.00
	Year	8	37.55	−1118.42	0.00
	Treatment	4	73.95	−1140.93	0.00
	Intercept	3	80.29	−1145.14	0.00

**TABLE 2:** Results of the top linear mixed models explaining variation in reproductive output in tree swallows showed a treatment effect on mean (A) clutch size, (B) egg volume, and (C) hatching success.

A	Clutch		
Predictors	Estimates	CI	*p*
(Intercept)	26.81	−15.39—69.01	0.212
Treatment (control)	−0.59	−0.97—‐0.22	**0.002**
ICC	1.00		
N_BoxID_	94		
Observations	224		
Marginal *R* ^2^	0.000		

B	Volume		
Predictors	Estimates	CI	*p*
(Intercept)	1625.96	1596.36–1655.56	**< 0.001**
Treatment (control)	44.51	22.35–66.66	**< 0.001**
Year (2014)	−19.10	−55.81 – 17.61	0.306
Year (2015)	307.39	271.50–343.2	**< 0.001**
Year (2016)	13.64	−30.96 – 58.24	0.547
Year (2017)	273.27	236.62–309.91	**< 0.001**
Year (2018)	262.85	216.57–309.14	**< 0.001**
N_BoxID_	82		
Observations	181		
Marginal *R* ^2^	0.795		

C	Hatching success		
Predictors	Estimates	CI	*p*
(Intercept)	79.61	67.25–91.97	**< 0.001**
Treatment (control)	−13.91	−22.45—‐5.36	**0.001**
Year (2014)	−5.92	−21.08—9.23	0.442
Year (2015)	−3.90	−18.60—10.80	0.601
Year (2016)	8.35	−8.28—24.97	0.323
Year (2017)	−7.10	−22.57—8.36	0.366
Year (2018)	14.08	−2.15—30.31	0.089
N_BoxID_	93		
Observations	217		
Marginal *R* ^2^	0.093		

*Note:* Bold values indicate significance at the *p* = 0.05 level.

For determining reproductive performance during only the 2017–2018 years, we used the same methods described above, but on a subset of 54 individuals across the two breeding seasons. In our analysis of clutch size for 2017 and 2018, the “treatment” model again ranked highest and carried the highest model weight (*w*_*i*_ = 0.44; Table 3A); calcium‐supplemented nests had statistically significantly larger mean clutch sizes compared to control nests (*p* < 0.001; Table 4A). Clutch size in the control nests was estimated to be smaller by 0.66 eggs (95% CI: −1.23, −0.09) compared to the calcium‐supplemented nests. In analyzing egg volume for 2017–2018, the “treatment × year” model ranked highest and carried the highest model weight (*w*_*i*_ = 0.97; Table 3B); calcium supplemented nests had larger mean clutch sizes compared to control nests (*p* < 0.001; Table 4B). Specifically, egg volume in the control nests was estimated to be smaller by 36.29 mm^3^ eggs (95% CI: −73.24, 0.66) compared to the calcium supplemented nests. In analyzing hatching success for 2017–2018, the “treatment × year” model ranked highest and carried the highest model weight (*w*_*i*_ = 0.36; Table 3C); calcium supplemented nests in 2018 had significantly higher hatching success compared to control nests (*p* = 0.001; Table 4C).

**TABLE 3:** Mothers at nests with supplemented calcium produced and hatched more and larger eggs, with some variation by year, as indicated by the model set and rankings exploring the importance of calcium supplementation (“Treatment”) and “year” on mean clutch size per nest, mean hatching success per nest, and mean egg volume per nest in Tree Swallows at a high‐elevation valley in northern Colorado during 2017–2018. The number of parameters (*K*), −2 log‐likelihood (−2LL), and model weights (*w*_*i*_) are shown for each model and the models are ranked by their AICc differences relative to the best model in the set (*∆*AICc_*i*_). The minimum AICc value for mean clutch size per nest was 482.53, for mean egg volume per nest it was 601.69, and for mean hatching success per nest it was 482.53. “Box” was included as a random effect in each model.

Dependent Variable	Model	*K*	*∆*AICc_*i*_	−2LL	*w*_*i*_
(A) Mean Clutch Size per Nest	Treatment	4	0	−109.39	0.44
	Treatment × year	6	11.96	−107.65	0.24
	Treatment + year	5	14.12	−109.33	0.15
	Intercept	3	18.41	−111.66	0.14
	Year	4	21.13	−111.75	0.04
(B) Mean egg volume per nest	Treatment × year	6	0	−277.84	0.97
	Treatment + year	5	6.92	−282.57	0.03
	Year	4	19.64	−290.15	0.00
	Treatment	4	20.41	−290.53	0.00
	Intercept	3	32.22	−297.61	0.00
(C) Mean hatching success per nest	Treatment × year	5	0	−236.01	0.36
	Year	4	0.74	−238.74	0.25
	Intercept	2	1.34	−240.15	0.18
	Treatment + year	4	2.36	−238.39	0.11
	Treatment	3	2.62	−239.68	0.10

**TABLE 4:** Results of the top linear mixed models explaining the variation in reproductive output in tree swallows showed an effect of experimental calcium treatment on mean (A) clutch size, (B) egg volume, and (C) hatching success for the 2017–2018 years.

A	Clutch		
Predictors	Estimates	CI	*p*
(Intercept)	5.15	4.73–5.58	**< 0.001**
Treatment (control)	−0.66	−1.23 to −0.09	**0.024**
ICC	0.17		
N_BoxID_	55		
Observations	69		
Marginal *R* ^2^/conditional *R* ^2^	0.074/0.231		

B	Volume		
Predictors	Estimates	CI	*p*
(Intercept)	1920.44	1894.33–1946.54	**< 0.001**
Treatment (control)	−36.29	−73.24–0.66	0.054
Year (2018)	62.52	15.75–109.29	**0.01**
Treatment (control) × year (2018)	−26.59	−94.28–41.10	0.434
ICC	0.19		
N_BoxID_	48		
Observations	54		
Marginal *R* ^2^/conditional *R* ^2^	0.246/0.387		

C	Hatching success		
Predictors	Estimates	CI	*p*
(Intercept)	87.37	80.52–94.21	**< 0.001**
Treatment (control)	12.05	2.90–21.20	**0.011**
Year (2018)	1.12	−6.58–8.81	0.772
Treatment (control) × year (2018)	−20.13	(31.98 to −8.27)	**0.001**
ICC	0.79		
N_BoxID_	46		
Observations	58		
Marginal *R* ^2^/conditional *R* ^2^	0.172/0.828		

*Note:* Bold values indicate significance at the *p* = 0.05 level.

### 3.2 Calcium Supplementation and Telomere Length

**FIGURE 2:**
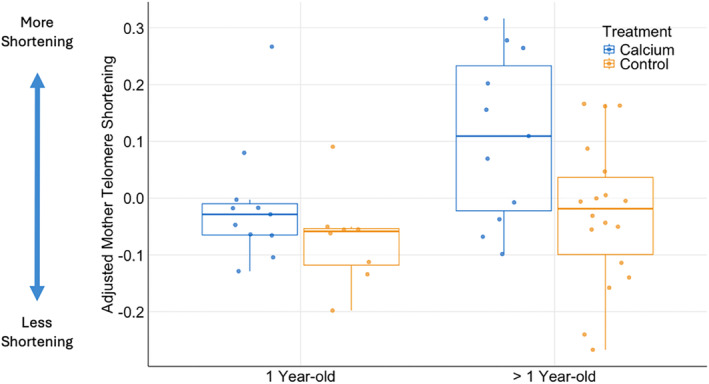
Female tree swallows supplemented with calcium showed increased telomere shortening as indicated by female telomere shortening adjusted for regression to the mean for calcium (blue; *n* = 22) versus control nests (yellow; *n* = 26). Older female tree swallows also showed increased telomere shortening as indicated by female telomere shortening adjusted to regression to the mean for 1‐year‐old (*n* =29) and older than 1‐year‐old birds (*n* =19). Adjustments for regression to the mean scales the mean of the data to zero.

In analyzing telomere length in mother tree swallows, the “Treatment + Age” model carried the highest model weight (*w*_*i*_ = 0.42; Table 5); supplemented nests had higher adjusted telomere shortening compared to controls (*p* = 0.006; Figure 2A and Table 6). Specifically, control mothers were estimated to have 0.11 lower adjusted telomere shortening (95% CI: −0.18, 0.03) compared to calcium‐supplemented nests. In looking at maternal age, one‐year‐old mothers had significantly lower adjusted telomere shortening compared to older mothers (*p* = 0.004; Figure 2B and Table 6). Specifically, one‐year‐old mothers were estimated to have −0.08 less adjusted telomere shortening (95% CI: −0.15, 0.00) compared to that of calcium supplemented mothers. We reran model selection with brood size in place of clutch size and the “Treatment + Age” model was again the top model (Table 7).

**TABLE 5:** Mothers at nests with supplemented calcium showed the most telomere shortening over the course of the breeding season as indicated by their high model weights and rankings among the set of models exploring the importance of calcium supplementation (“Treatment”), “Age,” “Year,” and clutch size (“Clutch”) on telomere shortening in breeding female Tree Swallows. Telomere length at time point one was included as a covariate in each model to account for baseline telomere length. qPCR plate was included as a covariate in each model. The number of parameters (*K*), −2 log‐likelihood (−2LL), and model weights (*w*i) are shown for each model and the models are ranked by their AICc differences relative to the best model in the set (∆AICc).

Model	*K*	∆AICc_*i*_	−2LL	*w*_*i*_
Treatment + age	6	0.00	35.81	0.42
Treatment × age	7	1.24	36.57	0.23
Treatment	5	2.09	33.46	0.15
Treatment + clutch	6	4.27	33.70	0.04
Treatment + year	6	4.71	33.46	0.03
Null model	4	5.55	30.48	0.03
Age	5	6.01	31.50	0.02
Clutch	5	6.02	31.51	0.01
Treatment × clutch	7	6.71	33.87	0.01
Age × clutch	7	6.93	33.76	0.01
Age + clutch	6	7.07	32.30	0.01
Age × year	7	7.12	33.63	0.01
Calcium × year	7	7.13	33.63	0.01
Year × clutch	7	7.63	33.41	0.01
Year	5	8.04	30.48	0.01
Age + year	6	8.57	31.53	0.00
Year + clutch	6	8.65	31.51	0.00

**TABLE 6:** Results of the top linear model explaining the variation in mother telomere shortening over the course of the breeding season showed an effect of experimental calcium treatment and age.

	Mother telomere shortening		
Predictors	Estimates	CI	*p*
(Intercept)	0.16	0.02 to 0.30	**0.029**
Treatment (control)	−0.11	−0.18 to −0.03	**0.006**
Age (1 year old)	−0.08	−0.15 to −0.00	**0.041**
Mother TL	−0.01	−0.05 to 0.03	0.748
Plate	−0.01	−0.02 to 0.00	0.156
Observations	48		
Marginal *R* ^2^/conditional *R* ^2^	0.245/0.175		

*Note:* Bold values indicate significance at the *p* = 0.05 level.

**TABLE 7:** Mothers at nests with supplemented calcium show the most telomere shortening during the breeding season as indicated by their high model weights and rankings among the set of models exploring the importance of calcium supplementation (“Treatment),” “Age,” “Year,” and brood size (“Brood”) on telomere shortening in breeding female Tree Swallows. Telomere length at time point one was included as a covariate in each model to account for baseline telomere length. qPCR plate was included as a covariate in each model. The number of parameters (*K*), −2 log‐likelihood (−2LL), and model weights (*w*_*i*_) are shown for each model and the models are ranked by their AICc differences relative to the best model in the set (∆AICc_i_).

Model	*K*	∆AICc_*i*_	−2LL	*w*_*i*_
Treatment + age	6	0.00	35.81	0.42
Treatment × age	7	1.24	36.57	0.23
Treatment	5	2.09	33.46	0.15
Treatment + year	6	4.71	33.46	0.04
Treatment + brood	6	5.36	33.16	0.03
Null model	4	5.55	30.48	0.03
Age	5	6.01	31.50	0.02
Calcium × brood	7	6.76	33.84	0.01
Brood	5	6.79	31.12	0.01
Age × year	7	7.12	33.63	0.01
Treatment × year	7	7.13	33.63	0.01
Age + brood	6	7.56	32.06	0.01
Age + brood	7	8.00	33.22	0.01
Year	5	8.04	30.48	0.01
Age + year	6	8.57	31.53	0.01
Year + brood	6	9.41	31.14	0.00
Year × brood	7	9.90	32.28	0.00

**FIGURE 3:**
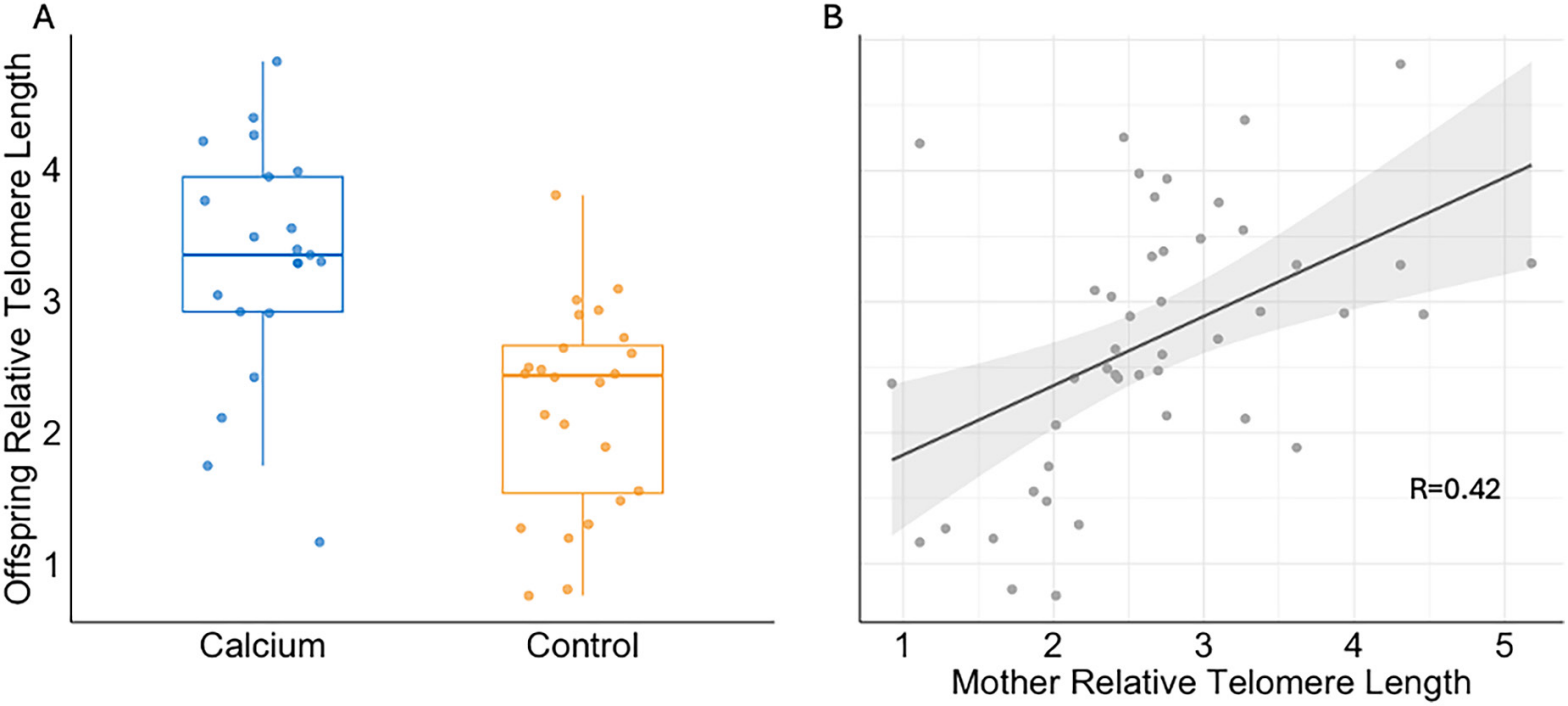
In 12‐day‐old nestling tree swallows, relative telomere length differs between calcium supplemented nests and control nests (A), and is highly correlated with mother telomere length pre‐breeding (B).

The “Treatment + Mother Pre‐breeding Relative Telomere Length” model carried the most model weight (*w*_*i*_ = 0.44; Table 8) for offspring RTL; calcium supplemented offspring had significantly longer average RTL per nest at 12 days compared to control chicks (*p* = 0.011; Figure 3A), with control nests having offspring with an estimated 0.63 shorter RTL (95% CI: −1.10, −0.15) compared to calcium supplemented nests (Table 9). In addition, offspring RTL was significantly positively correlated with mother pre‐breeding RTL (*p* = 0.011; Figure 3B), with an estimated correlation of 0.33 (95% CI: 0.08, 0.59).

**TABLE 8:** Relative telomere length (TL) of nestling Tree Swallows at 12 days old was dependent on whether their nest was supplemented with calcium (“Treatment”) and the relative telomere length of their mothers pre‐breeding as indicated by model weights and rankings for models exploring the importance of calcium supplementation on telomere length in nestling Tree Swallows. qPCR plate was included as a fixed effect in each model. “Box” was included as a random effect in each model. The number of parameters (*K*), −2 log‐likelihood (−2LL), and model weights (*w*_*i*_) are shown for each model and the models are ranked by their AICc differences relative to the best model in the set (*∆*AICc_*i*_).

Model	*K*	∆i	‐2LL	*w*_*i*_
Treatment + mother TL	6	0.00	−46.88	0.44
Treatment × mother TL	7	2.36	−46.66	0.13
Mother TL + year	7	2.63	−48.20	0.12
Treatment	6	3.95	−50.19	0.06
Mother TL	5	3.97	−50.20	0.06
Treatment + mother age	5	4.04	−48.90	0.06
Mother TL *year	6	5.10	−48.02	0.03
Treatment + year	7	5.55	−49.66	0.03
Treatment + mother age	6	5.86	−49.81	0.02
Treatment × mother age	7	6.16	−48.56	0.02
Mother TL × mother age	7	6.42	−48.68	0.02
Treatment × year	7	8.36	−49.65	0.01
Null model	4	13.35	−56.17	0.00
Year	5	14.04	−55.24	0.00
Mother age	5	14.26	−55.35	0.00
Mother age + year	6	15.74	−54.75	0.00
Mother age × year	7	18.46	−54.70	0.00

**TABLE 9:** Results of the top linear model explaining the variation in juvenile telomere length (TL) over the course of the breeding season showed an effect of experimental calcium treatment and mother telomere length.

	Juvenile telomere length		
Predictors	Estimates	CI	*p*
(Intercept)	1.45	0.48–2.42	**0.004**
Treatment (control)	−0.63	−1.10 to −0.15	**0.011**
Mother TL	0.33	0.08–0.59	**0.011**
Plate	0.10	0.04–0.16	**0.002**
*ICC*	0.03		
N_BoxID_	32		
Observations	45		
Marginal *R* ^2^/conditional *R* ^2^	0.515/0.527		

*Note:* Bold values indicate significance at the *p* = 0.05 level.

## 4 Discussion

During avian reproduction, parental investment contributes to higher levels of oxidative stress (Alonso‐Alvarez et al. 2004; Metcalfe and Alonso‐Alvarez 2010). When reproductive investment exceeds what is sustainable for parents, the costs to longevity are evidenced by accelerated telomere shortening (Sudyka et al. 2014). Indeed, studies in a range of organisms with experimentally increased brood size have shown that costly reproductive events have a negative impact on adult lifespan (Reichert et al. 2014) and the early life of offspring (Boonekamp et al. 2013; Herborn et al. 2014; Nettle et al. 2013). Traditionally, brood‐manipulation studies have reflected the relationship between investment in reproduction and telomere attrition; however, these studies did not account for investment in egg production, and telomere shortening was not specific to the breeding period. Measuring telomere length pre‐breeding facilitated assessment of telomere shortening during egg production, incubation, and nestling care. Here, we investigated the role that calcium supplementation plays in the relationship between reproductive investment and telomere length dynamics.

### 4.1 Calcium and Reproductive Parameters

Our results provide evidence that calcium is a limiting factor in tree swallow reproduction. Experimentally supplemented nests had higher reproductive productivity in the form of larger average egg volume, improved hatching success, and larger clutch size than control nests. While a variety of other studies have examined the effects of calcium supplementation on reproductive success in birds (e.g., Espín et al. 2016; Graveland and Berends 1997; Mänd and Tilgar 2003; Poulin and Brigham 2001; Tilgar et al. 2004; Wilkin et al. 2009), few have focused on the effect in areas where calcium is naturally available, and none have examined these effects in systems at elevations over 2000 m. Birds breeding at high elevation face a host of challenges that make living and breeding more metabolically costly than at lower elevations, resulting in relatively lower reproductive success with increasing elevation (Altshuler and Dudley 2006; Johnson et al. 2018; Nagy and Grabherr 2009). Although calcium supplementation appears to be advantageous in terms of reproductive output at our high‐elevation study site, the stress associated with increased reproductive effort comes at a direct cost to the mother, as evidenced by increased telomere shortening.

### 4.2 Calcium Supplementation and Telomere Shortening in Mother Tree Swallows

Mother tree swallows supplemented with calcium experienced increased telomere shortening compared to non‐supplemented control mothers. On average, supplemented females in our study also produced more, larger eggs, and hatched more eggs compared to control mothers. 0.86 additional chicks Supplemented females also and suffered more telomere shortening relative to controls., suggesting that producing and raising more offspring is costly in terms of stress and predicted lifespan.

The observed increase in telomere shortening may reflect a higher cost of breeding for supplemented mothers, likely due to the increased energy and resource expenditure that accompanies producing and raising more offspring. However, brood size was not included in the top model for telomere shortening, indicating that it was not necessarily the number of offspring produced, but rather, the calcium supplementation itself that resulted in brood sizes above what is optimal for each tree swallow mother.

Parents should ideally produce the number of offspring that maximizes their overall fitness, meaning that an individual should not produce the maximum number of offspring possible per year, but rather, the maximum number that simultaneously optimizes longevity (Krebs and Charnov 1974; Lack 1947). The optimum number of offspring has been studied widely and varies with parental condition, as well as with the environmental conditions present during reproduction (e.g., De Heij, Van den Hout, and Tinbergen 2006; Murphy et al. 2000; Pettifor, Perrins, and McCleery 2001). Many studies have concluded that reproductive costs are “investment‐dependent,” as they are only apparent once reproductive efforts have surpassed what parents were prepared to sustain (Reichert et al. 2014; Santos and Nakagawa 2012). This was true of our study system, as increased telomere shortening was the cost of reproduction that mother tree swallows experienced after exceeding their optimum clutch size.

Telomere shortening has previously been linked to reproductive investment via exposure to oxidative stress (Epel et al. 2004; Haussmann and Marchetto 2010; Wiersma et al. 2004; Von Zglinicki 2002). Because reproduction entails elevated levels of cellular replication, higher investments in reproduction are expected to increase an individual's exposure to oxidative damage through increased reactive oxygen species (ROS), or decreased investment into antioxidant defenses, both of which contribute to telomere shortening (Blount et al. 2016; Selman et al. 2012).

In addition to calcium supplementation, and as expected, age class was also an important factor in predicting telomere shortening during the breeding season, with older birds experiencing more telomere shortening compared to younger, one‐year‐old birds. Ouyang et al. (2016) also found that tree swallow reproductive output increased with age, and that this higher reproductive output coincided with shorter telomeres. Our results support the Terminal Investment Hypothesis, as older birds produced more and larger eggs compared to younger birds, perhaps due to the fewer chances they have to reproduce in the future (Clutton‐Brock 1984; Stearns 1992). In the context of life history theory, younger birds may lay smaller clutches and exert less energy to keep their probability of future survival and fecundity high (Godfray, Partridge, and Harvey 1991; Klomp 1970). Contrary to many studies that show telomere length shortens faster for larger individuals (e.g., Ringsby et al. 2015; Pepke et al. 2022; Sheldon et al. 2021), tarsus length was not included in the top model of our study, a finding likely due to calcium treatment and age having larger effects than body size in this system.

### 4.3 Calcium Supplementation and Telomere Length in Nestling Tree Swallows

The cost of reproduction in birds can manifest in terms of parental survival, future reproduction, and/or offspring survival. The cost of reproduction may be observed as an effect on offspring (i.e., early‐life telomere length), especially when parental effort is increased (Knowles, Wood, and Sheldon 2010; Linden and Møller 1989; Martin 2004; Pettifor, Perrins, and McCleery 2001; Sudyka et al. 2014; Voirin et al. 2023). Here, calcium supplementation and pre‐breeding RTL of the mother were the most important factors in determining offspring telomere length. Even though mothers in experimentally supplemented nests showed increased reproductive output at the cost of faster rates of telomere shortening, their offspring had longer average RTL compared to control chicks. RTL of chicks in supplemented nests at 12 days old (mean = 3.23 ± 0.15 SE) was longer compared to that of control nests (mean = 2.25 ± 0.17 SE).

It may be that calcium‐supplemented mothers bear the extra cost associated with increased reproductive investment, leaving their offspring to reap the developmental benefits of increased calcium. In other calcium supplementation studies that did not measure telomere length, offspring benefited from excess calcium, even when their parents experienced increased reproductive success. Dawson and Bidwell (2005) found that supplementing calcium to breeding tree swallows not only led to larger clutch sizes, but also had a positive effect on offspring growth rate. Similarly, Tilgar, Mänd, and Mägi (2002) found that supplementing nesting Great Tits with calcium also led to larger clutch and brood sizes, while simultaneously leading to higher mean tarsus lengths of offspring. Previous supplementation studies have credited increases in the number of offspring and offspring growth to an overall reduction in cost of reproduction. Here, however, we provide evidence to the contrary; calcium supplementation resulted in more telomere shortening in mothers breeding at our high‐elevation study site.

Costanzo et al. (2016) directly manipulated brood size and found that nestlings and parents in enlarged nests had shorter telomeres and displayed more telomere shortening compared to control and reduced nests, pointing to calcium as the reason for increased offspring growth and survival, even with increased nestling competition. In our study, supplemented mothers suffered the adverse effects of increased productivity as shortened telomeres. Concurrently, 12 day old offspring in calcium‐supplemented nests had longer telomeres compared to 12 day old offspring in control nests.

The RTL of chicks was also dependent on the pre‐breeding telomere length of the mothers. In birds, studies of telomere length inheritance have found stronger correlations between mothers and offspring than between fathers and offspring (Asghar et al. 2015; Horn et al. 2011; Reichert et al. 2015), such that maternal effects might provide better explanations for offspring telomere length. Interestingly, germline cells may be more vulnerable to telomere shortening than somatic tissues as they are especially susceptible to oxidative stress (Metcalfe and Alonso‐Alvarez 2010).

## 5 Conclusion

Many brood and clutch manipulation experiments have concluded that birds raising enlarged broods suffer the consequences and costs of reproduction via increased telomere shortening, and thus potential life shortening (Bauch, Becker, and Verhulst 2013; Reichert et al. 2014). However, many of these studies were cross‐sectional or assessed as yearly shortening of telomere length, while many longitudinal studies focused either on long‐lived species or used captive populations of short‐lived species.

In the current study, telomere length was measured in female tree swallows before and after breeding, allowing evaluation of telomere shortening exclusively during the breeding period, therefore our inferences are specific to the effects of calcium supplementation on breeding. Studying a wild, short‐lived avian species also facilitated better understanding of telomere length dynamics in birds with a fast pace of life under natural conditions. Calcium supplementation was employed as a novel means of testing the link between reproductive investment and telomere shortening. While previous calcium supplementation studies concluded that excess calcium increases reproductive output and simultaneously reduced the cost of reproduction, we found that supplemental calcium increased reproductive output at the expense of increased telomere shortening—and by extension shortened lifespan—in the supplemented mothers. While calcium‐supplemented tree swallow mothers bear the burden associated with increased reproductive investment, their offspring appear to reap the developmental benefits of increased calcium in that telomeres were longer in chicks from supplemented nests.

Our results provide additional support for using telomere length dynamics to elucidate constraints on life history trade‐offs. Future work should include telomere length assessment to better understand classic life history trade‐offs in various species and under differing environmental conditions.

## Author Contributions

**Marina D. Rodriguez:** conceptualization (lead), data curation (lead), formal analysis (lead), funding acquisition (lead), investigation (lead), methodology (lead), project administration (lead), visualization (lead), writing – original draft (lead). **Susan M. Bailey:** investigation (supporting), methodology (supporting), resources (equal), supervision (equal), writing – review and editing (equal). **Paul F. Doherty Jr.:** conceptualization (supporting), formal analysis (supporting), methodology (supporting), resources (supporting), supervision (equal), writing – review and editing (equal). **Kathryn P. Huyvaert:** conceptualization (equal), investigation (supporting), methodology (supporting), supervision (equal), writing – review and editing (equal).

## Conflicts of Interest

The authors declare no conflicts of interest.

## Data Availability

The data that support the findings of this study are openly available via Dryad at https://datadryad.org/stash/share/1OdeICdgS3M2zsfeCXJhS9cRq5nLhWjzxfD1WQLBgKQ.
